# Attitudes of primary care physicians toward prescribing buprenorphine: a narrative review

**DOI:** 10.1186/s12875-019-1047-z

**Published:** 2019-11-15

**Authors:** Dexter L. Louie, Mehret T. Assefa, Mark P. McGovern

**Affiliations:** Stanford University, Palo Alto, CA 94304 USA

**Keywords:** Buprenorphine, Primary care providers, Attitudes, Barriers, Stigma

## Abstract

**Background:**

The opioid epidemic is a major public health issue associated with significant overdose deaths. Effective treatments exist, such as the medication buprenorphine, but are not widely available. This narrative review examines the attitudes of primary care providers (PCPs) toward prescribing buprenorphine.

**Methods:**

Narrative review of 20 articles published after the year 2000, using the Consolidated Framework for Implementation Research (CFIR) to organize the findings.

**Results:**

Three of the five CFIR domains (“Intervention Characteristics,” “Outer Setting,” “Inner Setting”) were strongly represented in our analysis. Providers were concerned about the clientele associated with buprenorphine, diversion, and their self-efficacy in prescribing the medication. Some believed that buprenorphine does not belong in the discipline of primary care. Other barriers included philosophical objections and stigma toward substance use disorders. Notably, two studies reported a shift in attitudes once physicians prescribed buprenorphine to actual patients.

**Conclusions:**

Negative attitudes toward buprenorphine encompassed multi-layered concerns, ranging from skepticism about the medication itself, the behaviors of patients with opioid use disorders, and beliefs regarding substance use disorders more generally. We speculate, however, that negative attitudes may be improved by tailoring support strategies that address providers’ self-efficacy and level of knowledge.

## Introduction

In 2017, a total of 47,600 Americans died of an overdose involving an opioid, an increase of over 150% compared to just 18,515 in 2007 [1]. Rising deaths have fueled widespread recognition of an opioid epidemic characterized by synthetic drugs, cultural conflict and stigma, and an escalating need for improved access to treatments.

The opioid agonist pharmacotherapies of methadone and buprenorphine have been shown to be efficacious in the reduction of opioid use and overdose death among patients with an opioid use disorder (OUD) [2, 3]. Buprenorphine, a partial mu-opioid receptor agonist, is a first-line treatment for opioid dependence, both for detoxification purposes as well as maintenance therapy. Given the scope of the epidemic, the hope has been that primary care providers (PCPs) would be willing and able to incorporate buprenorphine into their armamentarium. Indeed, data from a recent study of nine Federally Qualified Health Centers (FQHCs) indicates that the integration of buprenorphine maintenance therapy into primary care settings (as opposed to addiction specialty centers) significantly improves primary care quality health-care indicators [4, 5].

The adoption of buprenorphine, however, has been sluggish. Although the number of United States (US) physicians who have received the waiver (X-license) to prescribe buprenorphine increased from 3737 to 22,198 between 2003 and 2012, a study found that a full 96% of states in 2012 still had opioid dependence rates higher than their buprenorphine treatment capacity rates [6]. Furthermore, some physicians who do possess the waiver simply decline to prescribe. Among physicians who had already received their X-license, only 28% in one study were actually prescribing [7].

The dearth of interest in prescribing buprenorphine, even among those who have already passed the legal and regulatory hurdles to do so, prompts curiosity into the attitudinal and cultural barriers against prescribing buprenorphine. Indeed, previous studies of physicians in general (including, but not limited to PCPs) have identified negative attitudes toward buprenorphine as a barrier to adoption [8]. Therefore, the aim of this narrative review was to focus exclusively on PCPs to determine if attitudes in primary care play a similar obstructive role.

## Methods

The authors chose a narrative review (as opposed to a systemic review) for this subject material. The concept of “attitudes” is wide-ranging, qualitative, subjective, and not easily encompassed by the traditional tools of systemic reviews, including statistical summarization, analyses of internal/external validity, and meta-analysis. As discussed by Collins and Fauser, narrative reviews allow for a broader scope than systemic reviews, which are often constrained by strict methodology [9]. This narrative review sought to utilize a liberal and inclusive search process to discern a coherent narrative thread to the concept of PCP attitudes toward buprenorphine and the related topic of buprenorphine adoption/implementation. The authors attempted to be broad and comprehensive, and therefore did not intend for this review to be either exhaustive or narrowly-defined.

The authors searched the following databases: PubMed, PsychINFO, Scopus, Google Scholar, and Web of Science. Search terms included the following phrases in a variety of combinations: “Buprenorphine”/“Suboxone;” “Attitudes;” “Barriers”/“Implementation”/“Adoption;” and “Primary Care”/“Family Medicine”/“PCPs.” The authors found additional studies by consulting the references and citations of relevant studies in a form of snowball sampling. Studies were included if they met the following criteria: peer-reviewed, published between 2000 and 2018, English-language, conducted in the US, and discussed PCP attitudes toward buprenorphine.

Multiple articles were reviewed for relevance by authors DL (psychiatrist licensed to prescribe buprenorphine) and MA (PhD in epidemiology with a background in implementation science). The search yielded a total of 61 articles, which were subsequently reviewed in depth. Articles were excluded if they solely described the attitudes of non-PCPs (for example administrators, patients, insurance companies, or psychiatrists) or if PCP attitudes were not one of the primary outcome measures. Of the 61 articles reviewed in-depth, 14 surveyed populations other than PCPs, 17 did not measure attitudes as a primary outcome, three were expert-opinion style reviews, and one was not from the US. Six articles were literature reviews that addressed attitudes, but focused on other topics (for example, barriers to buprenorphine implementation). Contributions from the latter were included in this review where relevant, but were not the focus of this article. Therefore, a total of 20 studies met the eligibility criteria. This study examines the findings of these 20 articles for a more focused review.

To analyze provider responses to buprenorphine and its adoption, we utilized the Consolidated Framework for Implementation Research (CFIR) by Damschroder et al., a determinant framework used in implementation science to identify barriers and facilitators to adoption [10]. A recent review identified CFIR as an “operational” framework amenable to detailed process instructions and the translation of theory into practice [11]. CFIR contains five major domains, “Intervention Characteristics,” “Outer Setting,” “Inner Setting,” “Characteristics of Individuals,” and “Process.” The first three domains, “Intervention Characteristics,” “Outer Setting,” and “Inner Setting,” were strongly represented in the studies we reviewed.

## Results

### Intervention characteristics

Providers sometimes worried about the effectiveness of buprenorphine, echoing 12-step philosophies which question the wisdom of medication-assisted treatment (MAT) over a strategy of complete abstinence [12]. Nevertheless, even though physicians were skeptical, the studies we reviewed rarely recorded instances of providers openly questioning the strength and quality of the evidence surrounding buprenorphine—in fact, some positively endorsed its benefits in treating OUD [13, 14]. Multiple studies, however, noted that providers were concerned about the complexity of safely prescribing buprenorphine [14–16].

The financial impact of buprenorphine was cited as a barrier by some studies. Providers worried about the projected cost to patients due to the shortage of insurance carriers covering the medication [13]. Remuneration for physicians was also an important barrier [15, 17, 18]. Molfenter and colleagues found that providers were concerned about dispensing to uninsured patients in a fashion that would be cost-neutral to the clinic [14]. The data was not unanimous however, with one study finding that only 28% of respondents reported anxiety over financial issues [7]. Other issues related to cost also appeared, including variance in coverage by insurers, “fail first” requirements, and time-consuming prior authorizations [15]. Unfortunately, concerns about cost were not found to be ameliorated by experience prescribing buprenorphine. Storholm and colleagues noted that concerns about the costs of buprenorphine to the clinic stayed consistent before and after implementation of a buprenorphine program [17]. Netherland and colleagues also found that “experts” in prescribing buprenorphine were significantly more concerned about adequate reimbursement than “novices” or non-prescribers [18].

Providers were also concerned about the types of patients that buprenorphine would attract. Patients with an opioid use disorder were described by some as “high maintenance” [19], “difficult” [15], and “unreasonably demanding” [16]. Respondents bluntly stated, “[we] don’t want these type of patients [in our clinics]” [12], and “doctors do not want to deal with this population” [14]. Some providers stated that they were not prescribers “by choice” [19], and in particular, multiple studies noted that they feared they would be opening up “a flood gate and [be]…overrun” [12] or be “inundated” [20] if they started prescribing buprenorphine [12, 17, 20, 21]. A full 33.8% of non-waivered physicians in one study stated that nothing would increase their willingness to prescribe buprenorphine, and logistic regression analysis pointed to fears of being inundated as the primary driver of this sentiment [20].

Diversion was also a concern among physicians, prescribers and non-prescribers alike [15, 20–22]. In one study, 25.7% of physicians who were non-prescribers of buprenorphine reported that they had not pursued a waiver due to fears of diversion, the second most commonly cited concern behind fears of patient inundation (29%) [20].

In two studies of PCP attitudes in healthcare settings where buprenorphine programs had recently been instituted, physicians went so far as to reject buprenorphine altogether. Providers stated that buprenorphine was “not aligned with clinic goals” [23], or “extra stuff,” and simply “not what we do here [in primary care]” [12]. Without disputing the evidence behind buprenorphine, these respondents stated that they believed buprenorphine to be alien to the realm of primary care, and rejected its place in the clinic. Interestingly, this did not appear to mean that providers were completely against addiction treatment; one of these studies reported that PCPs felt that providing buprenorphine was inconsistent with their clinic’s goals, but that providing counseling for substance use disorders (SUDs) was consistent [23].

### Outer setting

The “Outer Setting” domain has multiple constructs, but only “Patient Needs and Resources” was prominently represented in this sample. Multiple studies noted that many physicians expressed a consistent lack of interest in prescribing on the grounds that their particular communities/patient populations had little or no need for buprenorphine [11, 17]. However, these sentiments were not often followed by explanations. For example, one respondent was quoted as simply stating “We’ve discussed it. We don’t see that there is much need in our clinic” [13]. Other barriers, such as “Cosmopolitanism,” “Peer Pressure,” and “External Policy & Incentives” were not consistent themes in this review.

### Inner setting

“Culture” and “Implementation Climate” were major issues within the domain of “Inner Setting.” In terms of culture, studies noted that programs and physicians favoring a 12-step approach held cultural views which led them to be more skeptical of buprenorphine as a legitimate treatment option [12, 24, 25]. Examples of these views included the perception that buprenorphine and other forms of MAT are simply “replacing one addiction for another,” or the concern that buprenorphine actually makes detox too pleasant and therefore removes opioid users’ motivation to change [26]. These programs also question whether or not patients should permanently remain on maintenance medication, as opposed to using it for detoxification only [26]. This perspective is also present in the criminal justice system; studies have indicated that a basic level of involvement with drug courts, for example, also poses a barrier to buprenorphine adoption [15, 25].

Stigma, another function of culture, remains prevalent against patients with an OUD. A national study of 1010 PCPs largely in private practice found widespread suspicion of opioid users both personally and professionally, such that a vast majority of respondents were unwilling to have a person with prescription OUD marry into the family (79%), or work closely with them on the job (77%) [27]. Furthermore, a full 66% of respondents viewed people with prescription OUD as more dangerous than the general population. This same study found that larger proportions of physicians expressed negative attitudes toward people with OUD compared to the general public. Notably, these statistics were for people with prescription OUD, and were not inclusive of heroin use disorder, a condition which arguably suffers from even greater stigma.

“Implementation Climate” measures six items, including “Tension for Change,” “Relative Priority,” and “Compatibility,” and providers’ comments indicated issues with all of the aforementioned. In terms of “Tension for Change” and “Relative Priority,” some PCPs did not identify treatment for OUD as a major need in their patient population [14]. Even among those recognizing the need for OUD treatment, some physicians felt that they as individuals had few reasons to change. As one respondent noted, “we have nurtured a culture here that tends to use this available referral service with substance abuse, etc., rather than trying to handle that yourself” [13]. “Compatibility” was also a prominent issue, with reports of persistent anxiety that prescribing buprenorphine would lead to acquiring an unwanted reputation. For example, providers were concerned about being viewed as the local “source” of buprenorphine [19], or the town’s “addiction-treatment provider” [15, 21]—perhaps thereby fulfilling the fear of being overwhelmed by patients, but also transferring the stigma of OUD onto the clinic.

### Provider self-efficacy and level of knowledge

In our review, two CFIR factors which cannot be described as “attitudes” per se—provider self-efficacy and level of knowledge—were nonetheless highly relevant in that they appeared to influence provider attitudes toward buprenorphine in this sample.

Provider self-efficacy, the first factor, was a frequent concern. Physicians who had not prescribed buprenorphine before consistently reported a lack of confidence to treat OUD without further training [14–16]. In one study, the top resources cited by non-prescribers as most likely to increase their willingness to prescribe buprenorphine included: information about local counseling resources, being paired with an experienced provider, and receiving more CME courses on OUD [20]. Even among physicians who reported positive attitudes towards buprenorphine, few were actually prescribers—e.g., only 28% in one study, due to lack of institutional and psychosocial/mental health support [7]. Multiple studies found that providers in general (both prescribers and non-prescribers of buprenorphine) desired the ability to consult or refer difficult cases to specialists [18, 28]. As noted by one respondent, “they’re not trained in addictions in general; they didn’t get it in medical school, they take an eight hour course or look at a CD-ROM and I’m sure they are not going to feel that comfortable” [13].

Similarly, level of knowledge was a frequent theme, as suggested by significantly divergent beliefs about buprenorphine among prescribers and non-prescribers. Multiple studies reported that providers who did not prescribe buprenorphine were more likely to estimate a lower efficacy of buprenorphine than those who did prescribe [16, 19, 26]. Most notably, this gap between prescribers and non-prescribers existed even among addiction specialists: only 37% of sampled addiction specialists who did not prescribe buprenorphine described it as “very effective” for maintenance therapy, compared to 82% of those specialists who did prescribe [16]. Another study found that 91% of buprenorphine prescribers agreed with the statement: “my patients with opioid addiction would be satisfied with BMT [Buprenorphine Maintenance Therapy],” compared to only 35% of non-prescribers (*p* < 0.001) [19].

Differences in knowledge and experience also applied to diversion. For example, while non-prescribers of buprenorphine expressed fears of gaining poor reputations for being a universally-maligned “source” of diverted buprenorphine in the community [19], buprenorphine-prescribing physicians made a more nuanced distinction between patients who illicitly buy buprenorphine to treat OUD, versus those who sell it in order to gain funds to buy other drugs, e.g., heroin [15]. Similarly, a study of 72 buprenorphine-prescribing physicians endorsed the belief that diversion often represents treatment of withdrawal symptoms, as opposed to a worsening of the opioid epidemic [28].

### Rural vs urban

Our review included one study of rural physicians [21], one in a non-urban setting [19], and multiple in urban settings [23, 28–30]. Other studies were unrestricted by geography or did not specify. Given the restricted number of studies, particularly those direct comparing rural and urban physicians, it is difficult to draw many conclusions. However, in rural samples similar concerns existed including: mistrust, negative perceptions and attitudes, attraction of stigmatized populations to one’s practice, and lack of knowledge/time/interest [19, 21].

## Discussion

The studies cited suggest that attitudes remain a significant barrier to the adoption and implementation of buprenorphine into the routine practice of many PCPs. Three of the five CFIR constructs were associated with negative or skeptical attitudes, for example: against characteristics of buprenorphine itself (“Intervention Characteristics”), against the need for buprenorphine (“Outer Setting”), and against a culture that welcomes buprenorphine (“Inner Setting”).

As noted above, PCPs were worried about buprenorphine on a number of levels: as a complex medication, as a costly drug, as a divertable substance, and as attractive to the “wrong” type of clientele. Although providers did not question the evidence surrounding buprenorphine, they expressed the concern that buprenorphine might be, figuratively speaking, more trouble than it is worth. As one respondent stated, “I don’t know why a physician would want to get credentialed and then advertise to bring in patients that are likely to be more difficult than others to treat” [13]. Another physician disgruntled with his/her organization’s new buprenorphine program said, “[We are]…victimized and you are [going to] victimize me some more” [12]. In a study of waivered and non-waivered physicians, of the 87% who already possessed the wavier to prescribe buprenorphine, a full 33% had no desire to prescribe at all [20].

Logistical hurdles to adopting buprenorphine may not only include an 8 h training, but also (depending on the provider) adding the capability for urine toxicology testing, protocols/schedules for induction monitoring, and establishing referral relationships with specialists. Cost also appears to be a consistent issue, even for those who regularly prescribe buprenorphine [17, 18]. Combined with hesitations regarding change in clientele and risks of diversion, some might conclude that buprenorphine has no place within a primary care clinic [12, 23].

Presumably, proponents of this idea would suggest that patients with OUD seek care from addiction specialists or psychiatrists [18, 28], and that undoubtedly more providers trained in addictions are needed. However in a national survey of PCPs, 72% identified OUD as a “very serious” or “extremely serious” problem facing the US [27], and in another study 73% of family physicians felt it was their personal responsibility to treat OUD [19]. And yet, in the same study only 10% of those not currently prescribing were interested in doing so. Similarly, it is confusing that some PCPs claim that their patient populations have little-to-no need for buprenorphine [13, 17, 19]. The discrepancy between acknowledging a need and duty to treat on the one hand, and an unwillingness to become a buprenorphine prescriber on the other is concerning. One possible explanation is that some PCPs live and work in areas relatively untouched by the opioid epidemic and therefore, do not serve populations with this particular need. Other PCPs may agree that OUD is a major issue, but simply believe that it is beyond their field’s scope of practice, and that their “duty to treat” may only extend to making a proper referral. Finally, some PCPs may simply be reluctant, perhaps because of a mixture of personal and professional hesitations, to treat OUD with buprenorphine. As summarized by one PCP:

*“I'm happy for someone else to do it but if I were prescribing [MAT] I bet it would be more negative because it's a lot of work. It opens you up to having to go to court and this population tends to be squirrelly, they don't tend to be honest with you and then you have to do drug screens; and it's just, probably for me it's more work than it's worth in that I know I could refer to specialists who do just that and they could keep an eye on them really well”* [15].

These concerns about buprenorphine also exist within an ongoing culture of stigma against OUD, the existence of which our review unfortunately confirmed. Most striking is the information that anti-substance use disorder stigma may even be more severe among physicians [27]. Apart from the stigma describing patients with SUDs as difficult or demanding, the fears of attracting a “bad” clientele by prescribing buprenorphine also overlapped with concerns that the stigma of OUD would “rub off” on the clinic, ruining the physician’s reputation. Even when OUD is de-stigmatized for therapeutic purposes by communities such as Narcotics Anonymous, there exists an additional stigma against the use of MAT like buprenorphine and methadone [12, 24, 25]. Unfortunately, the existence of stigma against SUDs and MAT is not “breaking” news. These continue to be ongoing issues which both providers and patients alike struggle with.

### Impact of provider self-efficacy and level of knowledge

Provider self-efficacy and level of knowledge were identified as two relevant factors which may impact attitudes towards buprenorphine. Although no studies directly tested the impact of low provider self-efficacy on attitudes toward buprenorphine, we speculate that the former might cause physicians to see buprenorphine as more complex, difficult, or onerous. Similarly, having a lower level of knowledge about buprenorphine was associated with having poorer perceptions about the efficacy of the medication [16, 19, 26].

Fortunately, some data suggests that the very act of prescribing buprenorphine may be enough to change perceptions [17, 26]. Storholm and colleagues [17] piloted a buprenorphine program in two community clinics and assessed the attitudes of providers and staff at multiple time points during the intervention. Many of the barriers perceived as being “medium-to-large” prior to the intervention decreased in size to become “small-to-medium” after implementation, including: provider motivation to prescribe buprenorphine, provider perceived need for buprenorphine, provider self-efficacy, and provider concern over attracting too many patients with an OUD. In contrast, logistical barriers remained prominent, including appointment wait times and difficulty with electronic medical record registry integration. These findings suggest that post-implementation process, providers became less concerned with the clinical (as opposed to logistic) viability of buprenorphine.

Green and colleagues took this a step further by conducting a study of physician attitudes within two not-for-profit prepaid group-model integrated health plans, wherein logistical barriers to prescribing buprenorphine were heavily reduced [26]. Physicians in such health plans were salaried, and were explicitly exempt from business decisions such as how to obtain and store buprenorphine in clinic, or the type of clientele they should be attracting. Green and colleagues found that “champions”—physician leaders dedicated to encouraging their colleagues to prescribe buprenorphine—were critically important for adoption. They also found that the act of prescribing was enough to change some physicians’ minds, largely due to witnessing their patients improve on the medication. For some physicians, however, neither the presence of champions nor the experience of prescribing buprenorphine was enough: they remained disappointed and skeptical. The researchers concluded that “clinicians adopting a more moralistic approach, and clinicians pessimistic about treatment for opioid problems generally, were less likely to support use of buprenorphine.” In particular, Green et al. underscored clinicians who appeared “burned out” as being highly likely to remain skeptical of buprenorphine.

These findings, however, are partially counterweighted by a study by Ober and colleagues, in which implementing a buprenorphine and naltrexone program at a FQHC did not improve opinions about the effectiveness or ease of use of MAT [23]. Willingness to use buprenorphine or naltrexone did not change significantly. Nevertheless, physicians were still significantly more likely after the intervention to state that SUDs could be effectively treated in primary care settings.

### Changing attitudes

Attitudes toward buprenorphine continue to serve as barriers to adoption by PCPs. Based upon our analysis of the various cultural and attitudinal factors at play, as well as the role of provider self-efficacy and level of knowledge in shaping those attitudes, we offer the following four suggestions for proponents of buprenorphine.

First, our review suggests that while physicians have accepted the evidence surrounding buprenorphine, many still remain skeptical in ways that those with higher levels of knowledge and self-efficacy regarding buprenorphine are not. Therefore, proponents of buprenorphine might focus on assumptions and fears about the medication that are not related to its efficacy. Trepidation that buprenorphine will negatively change one’s patient population is one example: while understandable, this concern is not clearly borne out by the literature. In fact, Storholm and colleagues found that after initiating their OUD treatment program, the frequency of fears about attracting a large homeless population fell from 55 to 43%, and fears of attracting too many patients with an OUD fell from 67 to 29% [17].

Indeed, some physicians note that prescribing buprenorphine to patients with an OUD can be a very positive and fulfilling experience after hearing their patients’ “saved-my-life stories” and other positive narratives [12]. In one study, having another physician with a waiver in one’s practice was significantly associated with becoming a prescriber [7]. Therefore, a second strategy might consist of finding ways to encourage physicians to try prescribing buprenorphine and challenge their own fears. Recruiting “champions” to lead by example and help encourage uncertain clinicians to take the proverbial plunge is one strategy, as was done by Green and colleagues [26]. Other means of convincing providers to push the boundaries of their comfort zones may include: institution-wide programs to create a culture of buprenorphine prescribing, giving incentives to providers who prescribe, and initially limiting provider exposure to buprenorphine patients and then gradually increasing that number over time.

Third, in recognizing the lack of provider self-efficacy, not to mention the extra work and learning curve required of PCPs to become buprenorphine prescribers, proponents of buprenorphine can continue to provide strong support systems/resources with specialty referral systems. Literature exists on programs such as PCSS-MAT [31], Project Echo [32], and other “hub and spoke” systems [33] which already exist to address these concerns, but can be further improved and expanded.

Lastly, while the above strategies are intended for PCPs who feel reluctant about buprenorphine, these tactics may be insufficient for those who are wholly opposed on moral or philosophical grounds. Instead, a more realistic approach might involve admitting that buprenorphine is no panacea while simultaneously emphasizing that embracing buprenorphine need not mean abandoning 12 step ideals completely. The idea that suffering can serve as an impetus to change, that some hardship can make better people out of us all, and that abstinence is an excellent possible outcome, remain admirable and worthy concepts. If we were able to fuse these ideas with current buprenorphine treatment practices, we would be doing a great service to both our patients as well as OUD treatment more broadly.

### Limitations

There are limitations to this narrative review. First, no formal meta-analysis was attempted due to methodological heterogeneity among studies, including study design, survey instruments and interview guides, and standardized scoring/analysis. Second, there are intrinsic difficulties measuring complex and nuanced ideas (e.g., cultural attitudes) through surveys or interviews only. Third, the 20 studies were from the US only, making it difficult to generalize beyond this population. Fourth, as is befitting a narrative review, the conclusions drawn here are the interpretations made by the authors, based only on the presented data and selected quotations provided by the authors of the individual studies. Without access to all of the data, including transcripts, notes, etc.—a highly impractical feat to review such material—some gaps and assumptions must necessarily exist. Lastly, this article did not attempt to fully review non-attitudinal barriers to buprenorphine adoption, including technical, legal, and logistical barriers. These barriers may simultaneously reflect and impact existing cultural attitudes and beliefs, and are therefore an important part of the discussion. Further works may consider exploring the relationships and interplay between cultural attitudes and other barriers.

## Conclusion

The attitudes of medication prescribers remain a significant barrier to buprenorphine adoption and implementation nationwide. Attitudinal obstacles comprise many of the main CFIR domains, spanning characteristics of the intervention itself to the outer systems that organizations and clinics inhabit. Nevertheless, implementation strategies designed to scale up buprenorphine in routine practice settings suggests that cultural attitudes may be changed by experience and proper support. For MAT prescribers, their patients, and perhaps society as a whole, this is promising news.

## Data Availability

All data generated or analyzed during this study are included in the cited published articles.

## Abbreviations

CFIR: Consolidated Framework for Implementation Research
FQHC: Federally-Qualified Health Center
MAT: Medication-Assisted Treatment
OUD: Opioid-Use Disorder
PCP: Primary Care Provider
SUD: Substance Use Disorder
